# Dual Synergistic Tumor‐Specific Polymeric Nanoparticles for Efficient Chemo‐Immunotherapy

**DOI:** 10.1002/advs.202301216

**Published:** 2023-08-07

**Authors:** Jiajia Xiang, Kexin Liu, Hongxia Xu, Zhihao Zhao, Ying Piao, Shiqun Shao, Jianbin Tang, Youqing Shen, Zhuxian Zhou

**Affiliations:** ^1^ Zhejiang Key Laboratory of Smart Biomaterials and Key Laboratory of Biomass Chemical Engineering of Ministry of Education College of Chemical and Biological Engineering Zhejiang University Hangzhou 310027 China; ^2^ ZJU‐Hangzhou Global Scientific and Technological Innovation Center Zhejiang University Hangzhou 311215 China

**Keywords:** cancer drug delivery, chemo‐immunotherapy, combination therapy, immunogenic cell death, synergistic effect

## Abstract

Chemo‐immunotherapy has made significant progress in cancer treatment. However, the cancer cell self‐defense mechanisms, including cell cycle checkpoint and programmed cell death‐ligand 1 (PD‐L1) upregulation, have greatly hindered the therapeutic efficacy. Herein, norcantharidin (NCTD)‐platinum (Pt) codelivery nanoparticles (NC‐NP) with tumor‐sensitive release profiles are designed to overcome the self‐defense mechanisms via synergistic chemo‐immunotherapy. NC‐NP remains stable under normal physiological conditions but quickly releases 1,2‐diaminocyclohexane‐platinum(II) (DACHPt, a parent drug of oxaliplatin) and NCTD in response to the tumor acidity. NCTD inhibits protein phosphatase 2A (PP2A) activity to relieve cell cycle arrest and downregulates the tumor PD‐L1 expression to disrupt the programmed cell death‐1 (PD‐1)/PD‐L1 interaction, synergistically enhancing Pt‐based chemotherapy and immunogenic cell death‐induced immunotherapy. As a result, NC‐NP exhibits potent synergistic cytotoxicity and promotes T cell recruitment to generate robust antitumor immune responses. The dual synergism exhibits potent antitumor activity against orthotopic 4T1 tumors, providing a promising chemo‐immunotherapy paradigm for cancer treatment.

## Graphical abstract

An effective strategy is reported here to reverse the cancer defense mechanisms via tumor‐specific dual synergistic norcantharidin (NCTD)‐platinum (Pt) codelivery nanoparticles (NC‐NP). The nanoparticles remain stable under normal physiological conditions but quickly release Pt and NCTD in response to the endosomal pH, synergistically enhancing chemotherapy and immunotherapy. The dual synergism exhibits potent antitumor activity, providing a promising paradigm for cancer chemo‐immunotherapy.

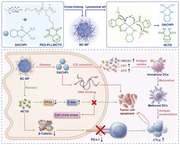

## 1 Introduction

Recent decades have witnessed the unprecedented success of immunotherapy in cancer treatment.^[^ ^1^ ^]^ However, the majority of patients fail to benefit from the present immunotherapies in the clinic, such as the immune checkpoint blockade (ICB) therapy, due to the heterogeneous tumor immune microenvironment^[^ ^2^ ^]^ — “Hot” tumors with massive infiltration of cytotoxic T lymphocytes (CTLs) respond substantially to the ICB therapy, while non‐CTL‐infiltrated “cold” tumors always show low response.^[^ ^3^ ^]^ Therefore, remodeling cold tumors into hot tumors remains top urgent for cancer immunotherapy.^[^ ^4^ ^]^

Increasing studies demonstrate that chemotherapy, in addition to killing tumor cells, can also modulate the immune system to elicit antitumor immune responses.^[^ ^5^ ^]^ Many chemotherapeutics, such as oxaliplatin (OXA),^[^ ^6^ ^]^ doxorubicin (DOX),^[^ ^7^ ^]^ and paclitaxel (PTX),^[^ ^8^ ^]^ can effectively induce immunogenic cell death (ICD) of tumor cells via immunostimulatory danger‐associated molecular patterns (DAMPs), including calreticulin (CRT),^[^ ^9^ ^]^ adenosine triphosphate (ATP),^[^ ^10^ ^]^ and high mobility group box 1 (HMGB1).^[^ ^11^ ^]^ The dying tumor cells release DAMPs as immunologic adjuvants to promote dendritic cell (DC) maturation and antigen presentation to T cells in lymph nodes,^[^ ^12^ ^]^ thereby activating the adaptive immunity.^[^ ^13^ ^]^ The ICD cascade process is capable of promoting intratumoral infiltration of CTLs,^[^ ^14^ ^]^ thus stimulating immunosuppressive cold tumors to immunogenic hot tumors.^[^ ^15^ ^]^ However, the efficacy of chemo‐immunotherapy is highly altered by the tumor cell self‐defense mechanisms,^[^ ^16^ ^]^ such as the cell cycle checkpoint‐mediated DNA repair,^[^ ^17^ ^]^ which causes cancer drug resistance and greatly compromises immune‐stimulatory ICD induction. Besides, T cells recruited by chemotherapy‐induced ICD would be badly abolished by the negative immune regulatory signaling pathways.^[^ ^18^ ^]^ Interferon‐γ (IFN‐γ) secreted by CTLs activates tumor cells to overexpress multiple immune checkpoints,^[^ ^19^ ^]^ such as programmed cell death ligand‐1 (PD‐L1).^[^ ^20^ ^]^ Moreover, chemotherapeutic drug‐treated tumor cells may upregulate PD‐L1 levels to escape immune surveillance of T cells, resulting in T cell dysfunction and exhaustion.^[^ ^21^ ^]^ Although the popular PD‐L1 blockade with peptide, antibody, or small‐molecule inhibitors momentarily interrupts programmed cell death‐1 (PD‐1)/PD‐L1 interactions,^[^ ^22^ ^]^ tumor cells can internalize the PD‐L1 and recycle them back to the cell surface.^[^ ^23^ ^]^ Thus, it is crucial to persistently downregulate the PD‐L1 expression to synergize the ICD‐induced immunotherapy.

The combination of chemotherapy with PD‐L1 downregulation has been shown to amplify the therapeutic benefits of ICD.^[^ ^24^ ^]^ Our group recently demonstrated the combination chemo‐immunotherapy of DOX and 5‐carboxy‐8‐hydroxyquinoline (IOX1, a histone demethylase inhibitor) through DOX‐triggered ICD and IOX1‐induced PD‐L1 downregulation in cancer cells, liberating the suppression of T cells and generating long‐term antitumor efficacy.^[^ ^24a^ ^]^ Nonetheless, cof chemotherapeutics and PD‐L1 downregulators is hurdled by their different and uncontrolled drug release profiles. An ideal drug delivery system to meet such requirements should be pretty stable under physiological conditions but quickly release the synergistic drugs responding to the tumor microenvironment to maximize the antitumor immune activities.

**Scheme 1:**
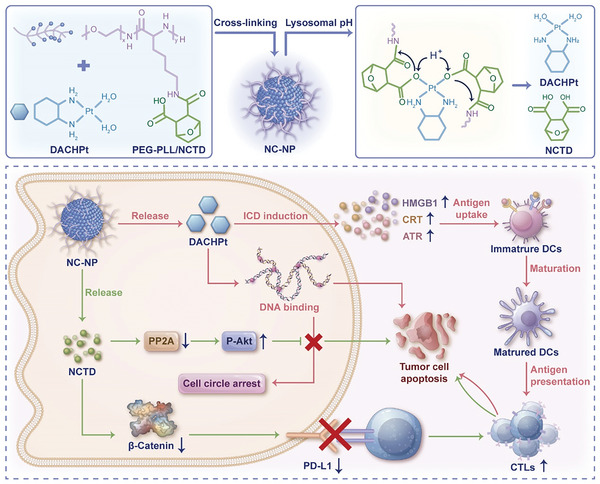
Schematic illustration of NCTD and OXA codelivery nanoparticles for cancer chemo‐immunotherapy. PEG‐PLL/NCTD coordinates with DACHPt, forming well‐defined nanoparticles (NC‐NP). Once accumulated in tumors, NC‐NP releases NCTD and Pt drugs in response to the tumor acidity. NCTD inhibits PP2A activity, depleting cell cycle arrest to reduce the DNA repair pathway and thus sensitize Pt drug‐based chemotherapy. DACHPt induces theICD of tumor cells to recruit CTLs, while NCTD downregulates the PD‐L1 expression of tumor cells and thus disrupts the PD‐1/PD‐L1 axis with CTLs, synergistically promoting antitumor immune responses.

Herein, we report the development of norcantharidin (NCTD)‐platinum (Pt) polymeric nanoparticles (NC‐NP) via the coordination between 1,2‐diaminocyclohexane‐platinum(II) (DACHPt, a parent drug of OXA) and NCTD conjugated poly(ethylene glycol)‐*b*‐polylysine (PEG‐PLL/NCTD) for a synergistic chemo‐immunotherapy (**Scheme** **1**). We have demonstrated that introducing β‐carboxylic acid amides in the Pt nanoparticles could endow tumor‐specific Pt drug release.^[^ ^25^ ^]^ The NC‐NP remains stable under normal physiological conditions but hydrolyzes rapidly to trigger the release of Pt and NCTD drugs in response to the acidic tumor intracellular compartment. NCTD efficiently inhibits protein phosphatase 2A (PP2A) activity, depleting cell cycle arrest to reduce the DNA repair pathway and thus sensitizing Pt drug‐based chemotherapy. DACHPt induces tumor cell ICD under the enhancement of NCTD to promote cytotoxic T lymphocyte (CTL) recruitment. At the same time, NCTD downregulates the tumor PD‐L1 expression via the β‐catenin pathway, counters Pt drug‐induced PD‐L1 overexpression, and disrupts the PD‐1/PD‐L1 interactions with CTLs, synergistically promoting the CTL activities and antitumor immune responses. These dual synergistic effects between NCTD and Pt drugs produce efficient antitumor activity against orthotopic 4T1 tumors, providing a promising chemo‐immunotherapy paradigm for cancer treatment.

## 2 Results

### 2.1 Preparation and Characterization of the NCTD and OXA Codelivery NC‐NP Nanoparticles

**Figure 1:**
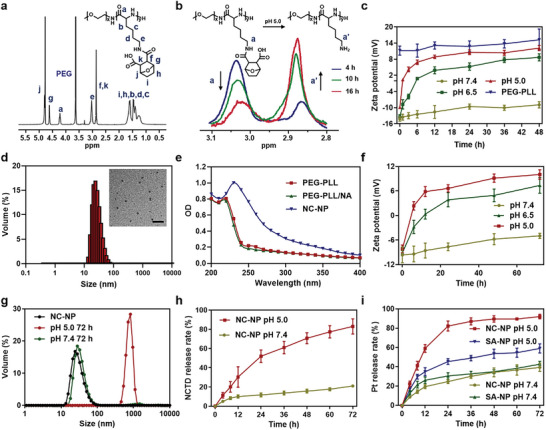
Physicochemical characterization and drug release of the NCTD and OXA codelivery NC‐NP nanoparticles. a) The ^1^H NMR spectrum of PEG‐PLL/NCTD. b) The hydrolysis of PEG‐PLL/NCTD at pH 5.0 detected by ^1^H NMR spectroscopy. c) The time‐dependent zeta potential changes of PEG‐PLL/NCTD at different pH. d) The size distribution of NC‐NP analyzed by DLS and the morphologies of NC‐NP observed by TEM, scale bar: 200 nm. e) UV–vis spectra of the solutions of PEG‐PLL, PEG‐PLL/NCTD, and NC‐NP. f) The time‐dependent zeta potential changes of NC‐NP at different pH. g) The size changes of NC‐NP after incubation at pH 7.4 or 5.0 for 72 h. h) The NCTD release profiles of NC‐NP at pH 7.4 or 5.0. i) The Pt drug release profiles of SA‐NP and NC‐NP at pH 7.4 or 5.0.

Linear PEG_114_‐PLL_76_ was synthesized as reported,^[^ ^26^ ^]^ followed by amidization with NCTD to obtain the carrier PEG‐PLL/NCTD. The ^1^H NMR spectrum of PEG‐PLL/NCTD indicated the complete amidization of amines (**Figure** **1**a). PEG‐PLL amidized with succinic anhydride (PEG‐PLL/SA) was synthesized as a control (Figure S1, Supporting Information). The acid‐triggered hydrolysis of β‐carboxylic acid amides in PEG‐PLL/NCTD was tracked by ^1^H NMR spectra at pH 5.0, indicated by the gradual decrease of the CH_2_NHC═O peak at *δ* 3.03 ppm, and the increase of the CH_2_NH_2_ peak at *δ* 2.86 ppm (Figure 1b). At pH 5.0, 34.6% and 73.2% of NCTD were released from PEG‐PLL/NCTD in 4 and 16 h, respectively, as calculated by the intensity change of these two peaks. The pH‐dependent hydrolysis of PEG‐PLL/NCTD was further confirmed by the zeta potential changes at different pH (Figure 1c). PEG‐PLL/NCTD remained in negative charge within 48 h at pH 7.4 but changed to be positively charged within 2 and 6 h at pH 5.0 and 6.5, respectively.

NC‐NP nanoparticles were obtained by complexing PEG‐PLL/NCTD with DACHPt. These NC‐NP nanoparticles had a diameter of ≈30 nm measured by dynamic light scattering (DLS) and a spherical morphology observed by transmission electron microscope (TEM) (Figure 1d). The UV spectrum of NC‐NP showed an absorption peak at a wavelength of 240 nm belonging to COO‐Pt bonding,^[^ ^27^ ^]^ indicating the successful complexation between PEG‐PLL/NCTD and DACHPt (Figure 1e). c to be 28.6%. SA‐NP with a similar size was also prepared as control nanoparticles by coordinating PEG‐PLL/SA with DACHPt (Figure S2, Supporting Information). NC‐NP remained negatively charged within 72 h at pH 7.4 and became positively charged within 6 and 12 h at pH 5.0 and 6.5, respectively (Figure 1f). Accordingly, NC‐NP kept stable under a neutral environment but collapsed in acidic conditions (Figure 1g; Figures S3 and S4, Supporting Information). Moreover, NC‐NP exhibited no significant hemolytic effects (Figure S5, Supporting Information), suggesting its good biocompatibility.

We next investigated the in vitro release of NCTD and Pt drugs from NC‐NP via a dialysis method in PBS at different pH. As shown in Figure 1h, NC‐NP released about 20% and 80% of NCTD in 72 h at pH 7.4 and 5.0, respectively. The pH‐dependent release of NCTD is due to the acid‐triggered hydrolysis of the β‐carboxylic acid amides.^[^ ^28^ ^]^ At pH 7.4, NC‐NP showed a slow and sustainable Pt release profile, similar to SA‐NP. At pH 5.0, NC‐NP released about 80% in 24 h, twice the SA‐NP (≈40%) at the same condition (Figure 1i). The results indicated that the pH‐responsive NCTD release would also accelerate the release of Pt drugs at acidic conditions.

### 2.2 Cellular Uptake and Cytotoxicity of NC‐NP

**Figure 2:**
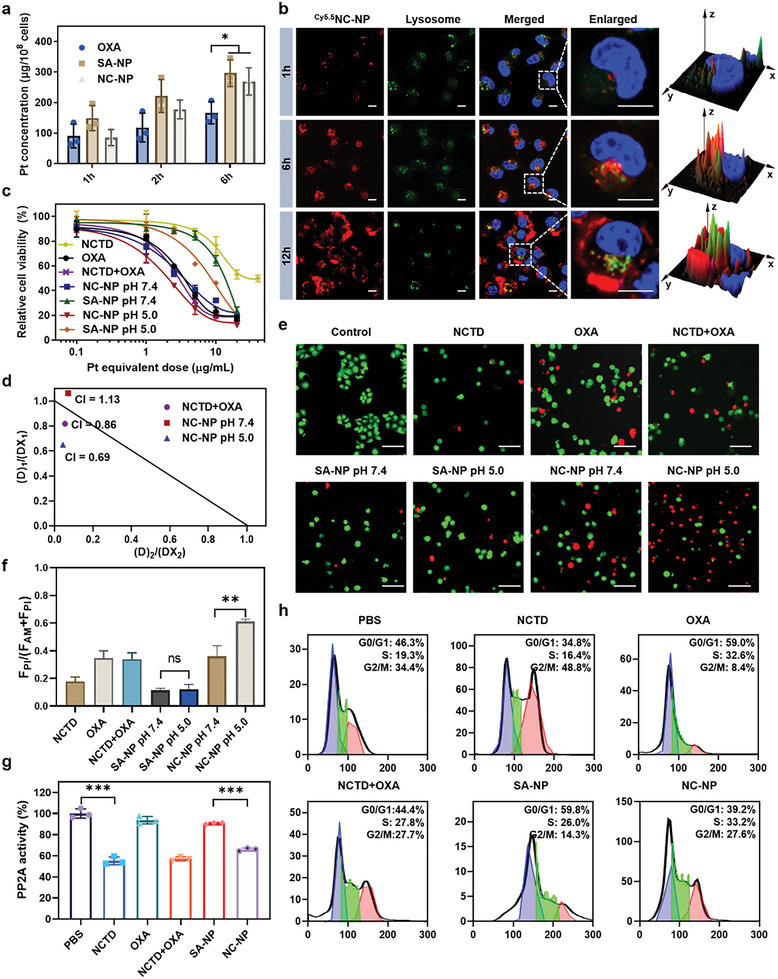
In vitro cytotoxicity and antitumor mechanism of NC‐NP. a) The time‐dependent intercellular Pt accumulation of OXA, SA‐NP, and NC‐NP in 4T1 cells measured by ICP‐MS. b) Subcellular distribution of NC‐NP observed by CLSM. 4T1 cells were cultured with ^Cy5.5^NC‐NP for 1, 6, or 12 h before observation (Cy5.5‐eq. dose, 0.5 µg mL^−1^). Nuclei stained with Hoechst 33342 are shown blue, lysosomes stained with LysoTracker are shown green, and ^Cy5.5^NC‐NP is shown red. Scale bars: 10 µm. c) The in vitro cytotoxicity of NC‐NP against 4T1 cells at pH 7.4 or 5.0 determined by MTT assay. d) The combination index (CI) of NCTD and OXA in NC‐NP. e) Confocal images of Calcein‐AM/PI‐stained 4T1 cells after treatments at different pH. SA‐NP or NC‐NP was pretreated at pH 7.4 or 5.0 for 24 h, followed by 48 h‐incubation with cells. Pt‐eq. dose of 3 µg mL^−1^, NCTD‐eq. dose of 5.2 µg mL^−1^. Calcein‐AM‐stained live cells are shown green, and PI‐stained dead/late apoptotic cells are shown red. Scale bars: 100 µm. f) Quantitative analysis of the live/dead cells in (c). g) PP2A activity of 4T1 cells after 6 h incubation with each formulation at an OXA eq. dose of 3 µg mL^−1^ and NCTD‐eq. dose of 5.2 µg mL^−1^. h) Flow cytometric analysis of cell cycle distribution of 4T1 cells after incubation with each formulation for 48 h. *p < 0.05; **p < 0.01; ***p < 0.001.

The cellular uptake rates of NC‐NP and SA‐NP were assessed by measuring the intracellular Pt concentrations using inductively coupled plasma‐mass spectrometry (ICP‐MS). NC‐NP and SA‐NP showed a time‐dependent internalization profile in 4T1 cells (**Figure** **2**a). At 6 h post‐treatment, the intracellular Pt concentration of 4T1 cells treated with NC‐NP (269.5 ± 45.0 µg Pt/10^8^ cells) or SA‐NP (296.4 ± 44.2 µg Pt/10^8^ cells) was much higher than those treated with OXA (166.2 ± 36.0 µg Pt/10^8^ cells). The internalization pathways of NC‐NP were studied in the presence of endocytic inhibitors (Figure S6, Supporting Information). The cellular uptake of NC‐NP was markedly suppressed at 4 °C, indicating an energy‐dependent route. Chlorpromazine, a clathrin inhibitor, dramatically reduced the cellular uptake of NC‐NP to 75%, indicating a clathrin‐associated mechanism. NC‐NP was further labeled with a fluorescent dye (Cy5.5), and their subcellular distribution was tracked by a confocal fluorescence microscope (Figure 2b). The intracellular Cy5.5 fluorescence increased with time, and most of the ^Cy5.5^NC‐NP was located in the lysosomes within 6 h. After 12 h, much red fluorescence appeared in the cytoplasm, indicating their capability to escape from the lysosome through the “proton‐sponge” effect.^[^ ^29^ ^]^

The cytotoxicities of NC‐NP against various cancer cell lines (4T1, A549, HeLa, B16, or HepG2 cells) were compared with multiple agents using an MTT assay (Figure 2c; Figure S7 and Table S1, Supporting Information). NC‐NP showed comparable cytotoxicity to OXA against 4T1 cell at pH 7.4 with a half‐maximal inhibitory concentration (IC_50_) of 3.22 µg mL^−1^, which was 4.2 times more potent than that of SA‐NP (13.52 µg mL^−1^). NC‐NP showed increased cytotoxicity (1.97 µg mL^−1^) after preincubation at pH 5.0, while OXA (3.03 µg mL^−1^) had no changes with the same treatment. NC‐NP also showed significantly enhanced cytotoxicity under acidic environments against B16, HepG2, A549, and Hela cells (Figure S8, Supporting Information). We further analyzed the synergistic effect of NCTD and Pt drugs in NC‐NP by employing the combination index (CI) at IC_50_ according to Equation (1), where CI > 1, CI = 1, and CI < 1 were regarded as antagonism, additivity, and synergism, respectively (Figure 2d; Figure S8 and Table S2, Supporting Information).^[^ ^30^ ^]^ At pH 7.4, NCTD and DACHPt in NC‐NP were antagonistic against the 4T1 (CI value of 1.13) cell line, additive against and Hela (CI value of 0.96) cell lines, but synergistic against B16 (CI value of 0.80), HepG2 (CI value of 0.64) and A549 (CI value of 0.79) cell lines. However, at pH 5.0, NCTD and DACHPt in NC‐NP were synergistic against all five cell lines, with CI values of 0.69, 0.34, 0.50, 0.52, 0.53 in the 4T1, B16, HepG2, A549, and Hela cell lines, respectively. The CI values of NC‐NP at pH 5.0 were remarkably lower than that of NCTD+OXA, which showed slight synergism or additivity against the five cell lines. The strong synergy between the two drugs in cancer cells under acidic conditions may result from rapid cell internalization and fast intercellular drug release.

(1) $$\begin{array}{c} \mathrm{CI}=\frac{{\mathrm{IC}}_{50}\left(\mathrm{NCTD}\;\mathrm{in}\;\mathrm{formulation}\right)}{{\mathrm{IC}}_{50}\left(\mathrm{NCTD}\right)}\;+\frac{{\mathrm{IC}}_{50}\left(\mathrm{OXA}\;\mathrm{in}\;\mathrm{formulation}\right)}{{\mathrm{IC}}_{50}\left(\mathrm{OXA}\right)} \end{array}$$

The Calcein‐AM/PI assay was adopted to further assess the pH‐dependent cytotoxicity of NC‐NP. As shown in Figure 2e, NC‐NP had comparable cytotoxicity to OXA at pH 7.4, and pretreatment at pH 5.0 further enhanced its cytotoxicity, higher than that of NCTD+OXA. In contrast, SA‐NP had low cytotoxicity at pH 7.4, while the acidic condition slightly increased its cell‐killing ability. Collectively, these results demonstrated the pH‐responsive cytotoxicity of NC‐NP. Further fluorescence analysis of the live and dead cells confirmed the pH‐responsive cytotoxicity of NC‐NP (Figure 2f).

After chemotherapy‐induced DNA damage, tumor cells can stimulate cell cycle checkpoints to arrest the cell cycle and start the DNA repair process.^[^ ^31^ ^]^ It is reported that NCTD can inhibit PP2A to stabilize MDM2 and downregulate p53, thus promoting cell cycle progression to accelerate cell apoptosis.^[^ ^32^ ^]^ Therefore, we performed the Ser/Thr phosphatase assay to determine the PP2A activity in 4T1 cells after each treatment. NC‐NP significantly decreased the PP2A activity by 35%, close to NCTD, while OXA and SA‐NP had no significant effect (Figure 2g). These results were verified in B16 cells (Figure S9, Supporting Information). To investigate the influence of PP2A inhibition on cell cycle progression upon acute DNA damage, we performed flow cytometry to analyze the cell cycle distributions of 4T1 cells at 48 h after each treatment (Figure 2h; Figure S11, Supporting Information). NCTD treatment decreased G0/G1 cell percentage and prominently promoted cell cycle to G2/M. OXA or SA‐NP treatment arrested cell cycle primarily at G0/G1 and S phase and significantly decreased G2/M phase cells with a percentage of 8.4% and 14.3%, respectively. The treatments with combination formulations, NCTD+OXA or NC‐NP, depleted the checkpoint arrest at G0/G1 phase and promoted 27.7% and 27.6% of cells to the G2/M phase, much higher than that of OXA and SA‐NP groups.

### 2.3 ICD Induction and PD‐L1 Downregulation of NC‐NP

**Figure 3:**
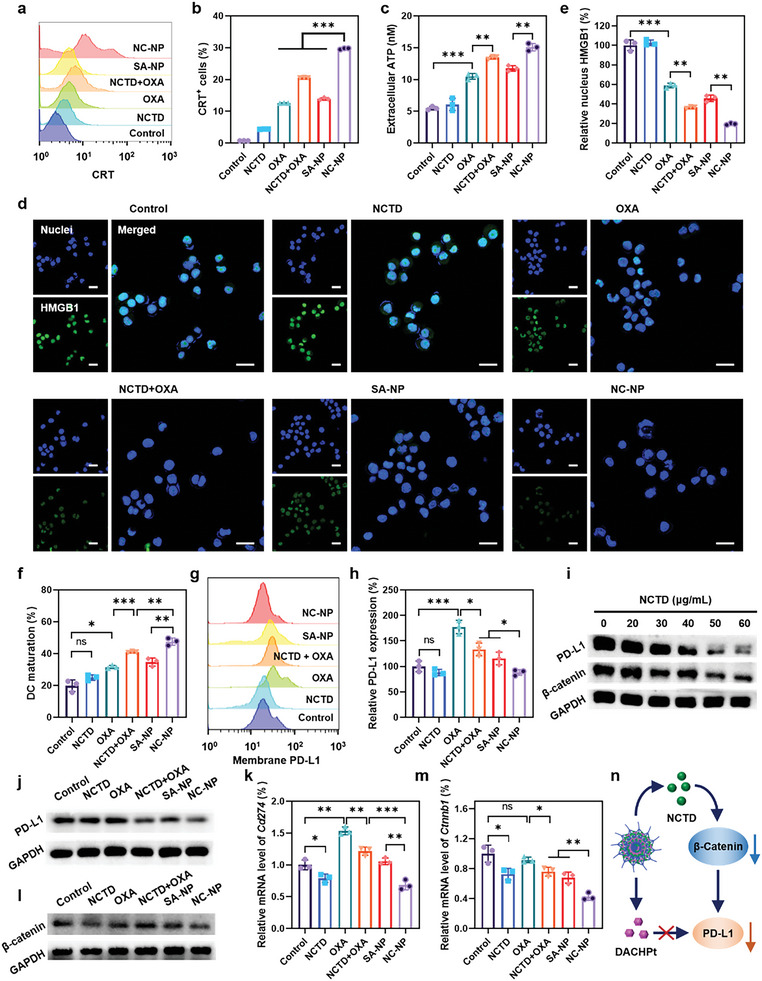
Tumor cell ICD and PD‐L1 downregulation induced by NC‐NP. a,b) Flow cytometric analysis (a) and quantification (b) of CRT‐positive cells; Pt‐eq. dose, 3 µg mL^−1^; NCTD‐eq. dose, 5.2 µg mL^−1^; 4 h incubation. c) Extracellular ATP release from 4T1 cells induced by NC‐NP; Pt‐eq. dose, 3 µg mL^−1^; NCTD‐eq. dose of 5.2 µg mL^−1^; 4 h incubation. d,e) CLSM images (d) and quantitative analysis (e) of the nucleus HMGB1 in 4T1 cells; Pt‐eq. dose, 3 µg mL^−1^; NCTD‐eq. dose, 5.2 µg mL^−1^; 24 h incubation. Nuclei stained with DAPI are shown blue, and HMGB1 is shown green. Scale bars: 25 µm. f) DC maturation analyzed by flow cytometry. 4T1 cells were cultured with different treatments for 24 h and then co‐incubated with BMDCs for another 24 h; Pt‐eq. dose, 3 µg mL^−1^; NCTD‐eq. dose, 5.2 µg mL^−1^. g,h) Flow cytometric analysis (g) and quantification (h) of membrane PD‐L1‐positive cells; Pt‐eq. dose, 3 µg mL^−1^; NCTD‐eq. dose, 5.2 µg mL^−1^; 24 h incubation. i) NCTD‐induced PD‐L1 and β‐catenin downregulation measured by western blotting. j) Endogenous PD‐L1 expression measured by western blotting. k) The PD‐L1 (Cd274) mRNA levels of 4T1 cells analyzed by qPCR; Pt‐eq. dose, 3 µg mL^−1^; NCTD‐eq. dose, 5.2 µg mL^−1^; 24 h incubation. l) Western blotting images of the β‐catenin in 4T1 cells with each formulation at a Pt‐eq. dose of 3 µg mL^−1^ and NCTD‐eq. dose of 5.2 µg mL^−1^; 24 h incubation. m) β‐catenin (Ctnnb1) mRNA levels analyzed by qPCR; Pt‐eq. dose, 3 µg mL^−1^; NCTD‐eq. dose, 5.2 µg mL^−1^; 24 h incubation. n) The schematic illustration of the mechanism of NCTD downregulating PD‐L1 expression. *p < 0.05; **p < 0.01; ***p < 0.001.

The ICD cascade events of cancer cells are featured by presenting DAMP molecules, including CRT, ATP, and HMGB1. We investigated the ICD‐inducing abilities of each formulation by measuring membrane CRT exposure, extracellular ATP secretion, and nucleus HMGB1 release. Flow cytometric analysis of CRT translocating to the cell membrane showed that NC‐NP induced 29.7% of CRT‐positive cells, significantly higher than that of OXA (12.6%), NCTD+OXA (20.5%), and SA‐NP (13.9%) (**Figure** **3**a,b). Secretion of ATP by tumor cells was evaluated using an ATP assay. The results showed that NC‐NP tripled extracellular ATP levels, with 1.4 and 1.3 folds higher than OXA and SA‐NP, respectively (Figure 3c). In addition, confocal images of nucleus HMGB1 showed that all the OXA‐containing formulations induced obvious nucleus HMGB1 release with an apparent decrease in HMGB1 fluorescence within nuclei (Figure 3d). Quantification of nucleus HMGB1 showed that NC‐NP dramatically caused an 81% reduction of HMGB1 fluorescence, a 1.5‐fold decrease compared to SA‐NP (Figure 3e). It is worth noting that NCTD alone did not induce tumor cell ICD while NCTD+OXA significantly increased ATP secretion, enhanced the amounts of membrane‐associated CRT, and dimmed the HMGB1 staining more efficiently than OXA alone. The result indicated that NCTD might potentiate OXA to induce tumor ICD. More importantly, NC‐NP caused ICD to a greater extent than NCTD+OXA, with 1.4‐, 1.2‐, and 1.3‐fold more efficient in ATP release, CRT translocation, and HMGB1 reduction, respectively. In contrast, SA‐NP showed comparable ICD‐inducing ability to OXA, as demonstrated by the similar ATP release, CRT translocation, and nuclear HMGB1 reduction. These results suggested that NC‐NP could efficiently induce ICD of tumor cells attributed to its fast cellular uptake and the potentiation of NCTD on OXA.

The DAMP molecules released in the ICD cascade are uptaken by antigen‐presenting cells (APCs), such as DCs, for recognition, processing, and presentation, thus activating the immune system.^[^ ^33^ ^]^ The in vitro DC maturation induced by tumor ICD was analyzed by determining the frequency of mature DCs (CD11c^+^CD80^+^CD86^+^) after culturing drug‐treated 4T1 cells with bone marrow‐derived DCs from BALB/c mice (Figure 3f; Figure S12, Supporting Information). The NCTD‐treated cells did not induce apparent DC maturation compared to the control. The frequency of mature DCs in OXA and NCTD+OXA treated cells was increased by 1.6‐ and 2.0‐fold to reach ≈32% and 41%, respectively. Notably, NC‐NP treated cells significantly promoted 48% of mature DCs, 1.5‐ or 1.4‐fold higher than OXA or SA‐NP.

Cancer cells overexpressing PD‐L1 will cause T cell exhaustion and thus an immunosuppressive tumor microenvironment, resulting in a low response rate to immunotherapy.^[^ ^34^ ^]^ Chemotherapy is always accompanied by PD‐L1 overexpression in tumor cells. As shown in Figure 3g,h, OXA treatment significantly upregulated the PD‐L1 expression by 1.8 folds in 4T1 cells. We found that NCTD could efficiently downregulate PD‐L1 in 4T1 cells in a dose‐dependent manner determined by western blotting (Figure 3i; Figure S13, Supporting Information); the PD‐L1 expression of 4T1 cells was markedly reduced to 51% by NCTD at a concentration of 50 µg mL^−1^. The NCTD+OXA and NC‐NP treated cells decreased PD‐L1 expression by 1.3‐ and 2.0‐fold compared to the control. The result was further confirmed by western blotting (Figure 3j; Figure S14, Supporting Information). The qPCR results also showed a decrease in PD‐L1 (Cd274) mRNA levels in the NCTD and NC‐NP treated cells (Figure 3k). In contrast, OXA‐treated cells showed an increase in Cd274 mRNA levels. These results suggested that NCTD could reverse the OXA‐induced PD‐L1 upregulation.

We further investigated the mechanism behind NCTD‐induced PD‐L1 downregulation. As shown in Figure 3i and Figure S13 (Supporting Information), NCTD also downregulated the β‐catenin expression in 4T1 cells in a dose‐dependent manner. The β‐catenin expression of 4T1 cells was reduced to 31% by NCTD of 60 µg mL^−1^. The western blotting assay showed that the β‐catenin expression in 4T1 cells was significantly decreased after the treatment of NCTD, NCTD + OXA, or NC‐NP (Figure 3l; Figure S14, Supporting Information). The qPCR results showed that the β‐catenin (Ctnnb1) mRNA levels in 4T1 cells were reduced to 72%, 68%, or 43% after the treatment of NCTD, NCTD+OXA, or NC‐NP, respectively (Figure 3m). It is reported that the downregulation of β‐catenin is associated with reducing PD‐L1 levels.^[^ ^24^, ^35^ ^]^ Thus, we proposed that NC‐NP released NCTD intracellularly to downregulate β‐catenin, which further induced the PD‐L1 reduction, reversing the released Pt drug‐caused PD‐L1 upregulation (Figure 3n).

### 2.4 In Vivo Antitumor Activity of NC‐NP

Subsequently, we compared the blood clearance kinetics of NC‐NP and SA‐NP with OXA. NC‐NP and SA‐NP showed similar and prolonged blood circulation time compared to OXA. At 24 h post‐injection, NC‐NP and SA‐NP had ≈6% of the initial dose remaining, while only 0.5% of OXA was left (Figure S15, Supporting Information). The blood clearance half‐life (T_1/2_) and area‐under‐curve (AUC) were calculated to further assess the pharmacokinetic properties (Table S3, Supporting Information). The elimination half‐lives (T_1/2_ β) and AUC of NC‐NP were 11.7 h and 299.2 ID%·h, which were 2.5‐ and 9.6‐fold higher than those of OXA (4.7 h, 31.0 ID%·h). The biodistributions of NC‐NP were investigated on 4T1 tumor‐bearing BALB/c mice. The NC‐NP treated mice had 3.1‐fold more tumor accumulation of Pt drugs than OXA at 24 h post‐injection (Figure S16, Supporting Information).

**Figure 4:**
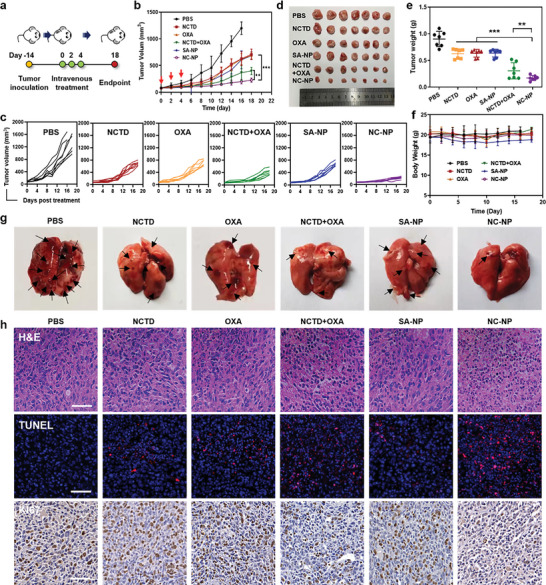
In vivo antitumor activity of NC‐NP. a) Schematic illustration of the experimental timeline and dosing schedule. Mice were inoculated with 5 × 10^5^ 4T1 cells, and the average tumor volume reached ≈100 mm^3^ after two weeks. The administration was initiated with i.v. injection of PBS, OXA, NCTD, NCTD + OXA, SA‐NP, or NC‐NP at a Pt‐eq. dose of 1 mg kg^−1^ and NCTD‐eq. dose of 1.7 mg kg^−1^ every two days three times. b) The tumor growth curves. c) The individual tumor growth curves in each group. d,e) Images (d) and average weights (e) of the dissected tumors. f) The changes in mice's body weight. g) Representative images of the dissected lungs at the experimental endpoint. h) H&E, TUNEL, and Ki67 staining of the dissected tumors. Scale bars: 50 µm. *p < 0.05; **p < 0.01; ***p < 0.001.

The in vivo antitumor activity of NC‐NP was evaluated in an orthotopic 4T1 tumor model in BALB/c mice. The mice were randomly divided into six groups (*n* = 7) when the tumors reached about 100 mm^3^ and intravenously treated with PBS, NCTD, OXA, NCTD+OXA, SA‐NP, or NC‐NP every other day three times (OXA‐eq. dose of 1 mg kg^−1^ and NCTD‐eq. dose of 1.7 mg kg^−1^) (**Figure** **4**a). OXA, NCTD, and SA‐NP moderately inhibited tumor growth compared to the PBS group during the treatment, and the tumors rebounded quickly after the treatments (Figure 4b,c). OXA+NCTD could stop the tumor growth within 10 days, and the tumors regained growth momentum after that. Notably, NC‐NP dramatically suppressed the tumor growth during the experiment, with a much higher tumor inhibition rate (81%) than those of OXA (30%), NCTD+OXA (66%), and SA‐NP (29%) (Figure 4b–e). The improved antitumor activity of NC‐NP was attributed to the enhanced tumor accumulation, pH‐responsive drug release, and synergistic effects of NCTD and OXA. Importantly, mice treated with NC‐NP had no significant body weight variation during the experiment, while OXA treatment caused a slight body weight decrease (Figure 4f). The histological analysis of normal tissues by H&E staining showed no noticeable histopathologic impairments in NC‐NP‐treated mice (Figure S17, Supporting Information). Orthotopic 4T1 tumors spontaneously metastasize primarily to the lung, leading to rapid deterioration. We thus investigated the anti‐metastasis ability of NC‐NP by counting the metastatic foci in the lung. Compared with the PBS group, NCTD, OXA, NCTD+OXA, and SA‐NP could reduce lung metastatic foci numbers, while NC‐NP could almost prevent lung metastasis (Figure 4g). The tumor tissues collected at the experimental endpoint sections were histologically analyzed using H&E, cell proliferation antigen (Ki67), and TUNEL staining (Figure 4h). The tumors' H&E and TUNEL staining revealed that NC‐NP treatment caused extensive cell apoptosis, and the Ki67 assay confirmed the cell proliferation‐inhibiting ability of NC‐NP. All the results suggested that the NCTD and Pt drugs codelivery strategy could effectively generate potent and synergistic antitumor therapeutic effects with low safety concerns.

### 2.5 In Vivo Immune Response Activation of NC‐NP

**Figure 5:**
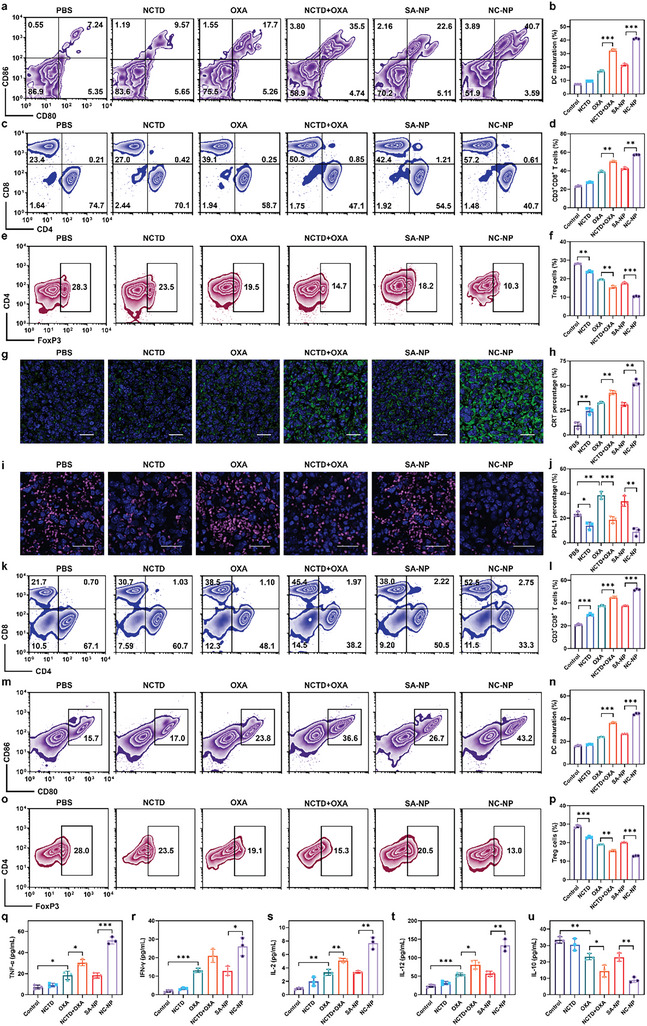
In vivo evaluation of NC‐NP induced antitumor immune responses. The 4T1 tumor‐bearing BALB/c mice with a tumor volume of ≈100 mm^3^ were treated with each formulation every other day three times; on 7 days after the last treatment, the TDLNs, tumors, and serum were collected for analysis. a–f) Flow cytometry analysis (a,c,e) and quantification (b,d,f) of the mature DCs (a,b), cytotoxic T lymphocytes (c,d), and regulatory T cells (e,f) in TDLNs. g–j) Evaluation of CRT exposure (g,h) and PD‐L1 expression (i.j) in the tumors by immunofluorescent staining (g,i) and ImageJ quantification (h,j). CRT stained with AF488 anti‐CRT is shown green, PD‐L1 stained with APC anti‐CD274 antibody is shown red, and the nuclei stained with DAPI are shown blue; Scale bars, 25 µm. k–p) Flow cytometry analysis (k,m,o) and quantification (l,n,p) of the cytotoxic T lymphocytes (k,l), matured DCs (m,n), and regulatory T cells (o,p) in the tumors. (q‐u) Quantification of systemic cytokines TNF‐ α (q), IFN‐ γ (r), IL‐2 (s), IL‐12 (t), and IL‐10 (u) in the serum of mice. *p < 0.05; **p < 0.01; ***p < 0.001.

To further clarify the mechanisms underlying the excellent antitumor activity of NC‐NP, we evaluated the capability of NC‐NP to induce tumor ICD in vivo and remodel the immune microenvironment (**Figure** **5**; Figure S18, Supporting Information). The 4T1 tumor‐bearing BALB/c mice with a tumor volume of ≈100 mm^3^ were treated with each formulation every other day for three injections, and 7 days after the last treatment, tumor‐draining lymph nodes (TDLNs) and tumor tissues were collected for immune cell analysis by flow cytometry. NC‐NP induced 41.0% of matured DCs in TDLNs, significantly higher than that of NCTD+OXA (32.3%) or SA‐NP (21.6%) (Figure 5a,b). The proportion of cytotoxic T lymphocytes (CTLs) was dramatically increased (Figure 5c,d), while the population of regulatory T lymphocytes (Tregs) was remarkably reduced (Figure 5e,f) in the NC‐NP group compared to those of other groups.

The immunofluorescence analysis of tumor tissues showed that the tumors treated with NC‐NP had the highest CRT exposure, 1.2‐ and 1.7‐fold of the NCTD+OXA or SA‐NP group (Figure 5g,h). NC‐NP‐treated tumors had the lowest PD‐L1 expression, just 39% or 27% of the NCTD+OXA or SA‐NP‐treated tumors, and even lower than the PBS group (Figure 5i,j). CTL infiltrating the tumors is a crucial prerequisite to activating antitumor immune responses.^[^ ^36^ ^]^ Therefore, we performed the flow cytometric analysis of the intratumoral infiltration of CTLs (Figure 5k). The frequency of infiltrated CTLs in NC‐NP‐treated tumors increased to as high as 51.8%, which was 1.2‐ and 1.4‐fold of the NCTD+OXA and SA‐NP groups, respectively (Figure 5l). Tumor‐infiltrating DCs can increase the antitumor activity of CTLs via the secretion of cytokines such as IFN‐γ and IL‐12. The flow cytometric analysis revealed that NC‐NP significantly enhanced the population of intratumoral matured DCs by 1.2 folds or 1.6 folds compared with NCTD+OXA or SA‐NP, respectively (Figure 5m,n). Moreover, NC‐NP significantly reduced the population of immunosuppressive Tregs in tumors to 12.9%, which was only about 4/5 or 3/5 of that in the NCTD+OXA or SA‐NP group (Figure 5o,p), possibly due to the pro‐apoptotic effect of NCTD on Tregs which has been reported recently.^[^ ^37^ ^]^ Together, NC‐NP exhibited superior efficacy in enhancing the intratumoral infiltration of antitumor lymphocytes and reducing immunosuppressive lymphocyte infiltration.

The level of systematic circulating cytokines, including immunoactivating TNF‐α, IFN‐γ, IL‐2, IL‐12, and immunosuppressive IL‐10, were measured using enzyme‐linked immunosorbent assay (ELISA). The NC‐NP‐treated mice had significantly enhanced levels of TNF‐α, IFN‐γ, IL‐2, and IL‐12, which were 2.8‐, 2.0‐, 2.3‐, and 2.3‐fold compared to the SA‐NP group, respectively (Figure 5q–t). By contrast, the immunosuppressive IL‐10 levels were dramatically decreased by the NC‐NP treatment, which was only 63% of NCTD + OXA and 39% of SA‐NP treatment (Figure 5u). The above results demonstrated that NC‐NP efficiently remodeled the tumor immune microenvironments and produced robust antitumor immune responses.

## 3 Discussion

Chemo‐immunotherapy has made significant progress in cancer treatment, but its therapeutic efficacy in solid tumors is greatly hindered by the cancer cell defense mechanisms, especially the cell cycle checkpoint and PD‐L1 upregulation. Therefore, exploiting new strategies to overcome the cancer defense mechanism represents the utmost challenge for cancer chemo‐immunotherapy. In this work, we demonstrated an effective strategy to combat cancer self‐defense via tumor‐specific dual synergistic polymeric nanoparticles from coordination between DACHPt and NCTD conjugated PEG‐PLL. The acid‐activated NC‐NP nanoparticles enabled robust dual synergistic mechanisms of chemosensitization and immunoenhancement with tumor selectivity.

While chemotherapeutics such as Pt drugs cause DNA damage to induce cell apoptosis, cell cycle checkpoint‐related DNA damage repair mechanisms significantly impair tumor cell‐killing abilities of drugs and provoke drug resistance of tumor cells, thereby compromising the therapeutic efficacies.^[^ ^31a^ ^]^ To tackle this issue, combining chemotherapy with strategies to inhibit DNA damage repair has been widely investigated in preclinical and clinical studies. However, the outcomes are far below expectations due to a deficiency in tumor selectivity. In the stable yet tumor acidity‐labile NC‐NP, NCTD not only acted as a combination therapeutics but also served as an acid‐cleavable linkage for Pt drug complexation, which enabled the tumor‐specific and simultaneous release of synergistic drugs in acidic lysosomal conditions, laying the foundation of collaborative treatments. NCTD is reported to inhibit the activity of PP2A, a major cell cycle regulator, followed by MDM2 upregulation and p53 downregulation,^[^ ^32a^ ^]^ thus depleting cell cycle arrest and sensitizing DACHPt‐induced cell apoptosis.

Some chemotherapeutic agents can elicit tumor cell ICD through the release of DMAPs to promote DC maturation, cross‐present tumor‐specific antigens, and prime CD8^+^ CTL response.^[^ ^38^ ^]^ However, immune checkpoints such as PD‐L1 are upregulated after chemotherapy or IFN‐γ action,^[^ ^39^ ^]^ drastically abolishing the functions of CTLs and accelerating T‐cell exhaustion. Codelivery of DACHPt and NCTD efficiently stimulated tumor ICD to elicit tumor immunogenicity for CTL priming and infiltration and downregulated the PD‐L1 levels in tumor cells via a β‐catenin‐PD‐L1 pathway simultaneously, thus turning an immune‐suppressive tumor into a T cell‐inflamed tumor and strengthening CTL‐mediated antitumor immune responses. Moreover, it has been reported that PP2A inhibition could convert cold microsatellite‐stable into microsatellite‐instable tumors, which triggered neoantigen generation, enhanced CTL infiltration, and inhibited Treg infiltration,^[^ ^40^ ^]^ further pointing to the potential synergisms of the PP2A inhibition effect of NCTD in NC‐NP.

## 4 Conclusions

In summary, we developed codelivery nanoparticles of DACHPt and NCTD with dual synergistic effects for cancer chemo‐immunotherapy. NC‐NP released the loaded NCTD and Pt drugs in a pH‐dependent manner due to the hydrolysis of acid‐labile β‐carboxylic acid amides. NCTD could promote cell cycle progression and deplete Pt drug‐induced checkpoint arrest, thus synergizing Pt drug‐based chemotherapy. Moreover, DACHPt efficiently induced tumor cell ICD and remodeled the immunosuppressive tumor microenvironment, including inducing DC maturation, promoting T cell infiltration, and facilitating antitumor cytokine secretion. NCTD also effectively reversed the Pt drug‐induced high PD‐L1 expression, disrupting the PD‐1/PD‐L1 axis and thus activating the antitumor immune responses. As a result, NC‐NP exhibited enhanced antitumor efficacy in the orthotopic 4T1 tumor model. Therefore, this polymeric nanoparticle codelivery system may be of great promise for cancer chemo‐immunotherapy.

## Conflict of Interest

The authors declare no conflict of interest.

## Supporting information

Supporting Information

## Data Availability

The data that support the findings of this study are available in the supplementary material of this article.
