# Supplementary material for: Dual Synergistic Tumor‐Specific Polymeric Nanoparticles for Efficient Chemo‐Immunotherapy
Source: Adv Sci (Weinh). 2023 Aug 7;10(29):2301216. doi: 10.1002/advs.202301216 (PMC10582463; doi:10.1002/advs.202301216)
Supplement: Supplementary file 1 — Supporting Information [file ADVS-10-2301216-s001.pdf]

ADVANCED SCIENCE
Open Access

Supporting Information

for *Adv. Sci.*, DOI 10.1002/advs.202301216

Dual Synergistic Tumor-Specific Polymeric Nanoparticles for Efficient Chemo-Immunotherapy

*Jiajia Xiang, Kexin Liu, Hongxia Xu, Zhihao Zhao, Ying Piao, Shiqun Shao, Jianbin Tang, Youqing Shen and Zhuxian Zhou**

Dual Synergistic Tumor-Specific Polymeric Nanoparticles for Efficient Chemo-Immunotherapy

*Jiajia Xiang†, Kexin Liu†, Hongxia Xu, Zhihao Zhao, Ying Piao, Shiqun Shao, Jianbin Tang, Youqing Shen, Zhuxian Zhou**

Prof. J. Xiang, K. Liu, Dr. H. Xu, Z. Zhao, Prof. S. Shao, Prof. J. Tang, Prof. Y. Shen, Prof. Z. Zhou
Zhejiang Key Laboratory of Smart Biomaterials and Key Laboratory of Biomass Chemical Engineering of Ministry of Education, College of Chemical and Biological Engineering, Zhejiang University, Hangzhou 310027, China.
E-mail: zhouzx@zju.edu.cn (Z. Zhou)
Prof. J. Xiang, Prof. S. Shao, Prof. J. Tang,
ZJU-Hangzhou Global Scientific and Technological Innovation Center, Hangzhou 311215, China.
† These authors contributed equally to this work.

Materials

Norcantharidin, oxaliplatin (OXA), and succinic anhydride were purchased from Energy Chemical Co., Ltd. (Shanghai, China). 3-(4,5-Dimethylthiazolyl-2)-2,5-diphenyltetrazolium bromide (MTT) and PP2A Immunoprecipitation Phosphatase Assay Kit were purchased from Sigma-Aldrich (Shanghai, China). LysoTracker Green and Hoechst 33342 were purchased from Invitrogen. RNase A was purchased from Solarbio (Beijing, China). ATP detection kit, Live and Dead Cell Double Staining Kit, and β-Catenin Mouse Monoclonal Antibody were obtained from Beyotime Inc. (Shanghai, China). CRT and anti-HMGB1 antibodies were purchased from Abcam Co. (Cambridge, UK). APC-CD11c, PE anti-CD80, FITC anti-CD86, PerPC anti-mouse CD3, FITC anti-mouse CD4, PE anti-mouse CD8a, PE anti-FOXP3 antibodies, and APC anti-mouse CD274 Antibody were purchased from BioLegend. Other reagents were purchased from Sinopharm

Chemical Reagent Co. Ltd. (Shanghai, China). PEG-PLL_{76} was synthesized according to the reported method, and the polymerization was initiated by the terminal amino group of α-Methoxy-ω-aminopoly (ethylene glycol; CH_3O-PEG-NH_2; Mw = 5,000).

Cell lines and animals

A549 (human lung adenocarcinoma cells), Hela (human cervical carcinoma cells), 4T1 (murine breast cancer cells), B16F10 (murine melanoma cells), and HepG2 (human hepatocellular carcinomas) were purchased from ATCC (American type culture collection). All tumor cells were cultured in RPMI-1640 medium (GIBCO Invitrogen Corporation/Life Technologies Life Sciences) or Dulbecco's Modified Eagle's Medium (DMEM) that containing 10% fetal bovine serum (FBS, Zhejiang Tianhang Biotechnology) and 1% penicillin/streptomycin (Invitrogen, Carlsbad, CA) in a humidified atmosphere containing 5% CO_2 at 37 °C.

BALB/c nude mice (6-8 weeks) were purchased from Shanghai Shrek Experimental Animals Co., Ltd. and kept in the standard clean-grade (SPF) shielding environment of the Laboratory Animal Center of Zhejiang Academy of Medical Sciences. All the animal procedures were conducted with the approval of the Animal Care and Use Committee of Zhejiang University (License number: 17611).

Synthesis of PEG-PLL/NCTD

PEG_{114}-PLL_{76} (100 mg, 0.23 mmol -NH_2) was dissolved in deionized water, and the pH of the solution was adjusted to 8.5. Norcantharidin (516 mg, $C_{NCTD}/C_{NH2} = 10$) was added portion-wise with stirring, while NaOH (0.1 N) was added to keep the pH around 8.5. After addition, the reaction mixture was stirred for another 2 h and then dialyzed against water (MWCO 3,500). After lyophilization, PEG-PLL/NCTD was obtained as a white solid (120 mg, yield 64%).

Preparation of NCTD and DACHPt co-delivery nanoparticle (NC-NP)

$DACHPtCl_2$ (5 mg/mL) was suspended in DI water and mixed with silver nitrate ($AgNO_3$:DACHPt=1:1) to form an aqueous complex. The mixture was then shaken (200rpm) at 25°C in the dark for 24 h. The white AgCl precipitates were removed by centrifugation, and the supernatant was then filtered (0.22 μm) to give hydrated

1,2-diaminocyclohexane-platinum (II) (DACHPt).

PEG-PLL/NCTD or PEG-PLL/SA were dissolved in DI water (C_{COOH} = 5 mM), and the solution pH was adjusted to 8.5 using NaOH (0.1 N), followed by the addition of DACHPt (C_{Pt} : C_{COOH} = 1: 1). The reaction was performed at 37°C for 72 h with shaking. The product was purified by dialyzing (MWCO 3,500) against DI water to remove the free platinum drug, and the resulting PEG-PLL/NCTD-DCHAPt (NC-NP) and PEG-PLL/SA-DCHAPt (SA-NP) nanoparticles were obtained. The size distributions were measured by dynamic light scattering (DLS) (Malvern Instrument Ltd., UK). The morphology of the nanoparticles was observed using a transmission electron microscope (Hitachi h-7000 TEM system, Kyoto, Japan). The Pt content was determined by inductively coupled plasma mass spectrometry (ICP-MS) (Perkin Elmer Optima 3100XL).

pH responsiveness of nanoparticles

The hydrolysis of PEG-PLL/NCTD was monitored using ^{1}H-NMR spectroscopy. Briefly, PEG-PLL/NCTD was dissolved in D_2O at a concentration of 10 mg/mL, and the solution pH was adjusted to 5.0 using DCl. The mixture was incubated at 37°C with shaking (200 rpm). At timed intervals, 600 μL of the solution was taken out for ^{1}H NMR characterization after the pH was adjusted to 8.5.

PEG-PLL/NCTD or NC-NP was dissolved in HEPES solution (10 mM) at pH 7.4, 6.5, or 5.0 and incubated at 37°C with shaking (200 rpm). At timed intervals, the solution was sampled for zeta potential measurement using DLS.

The stability of NC-NP in response to acidic conditions was measured by monitoring the size change. NC-NP was incubated in PBS at pH 7.4 or 5.0. at 37°C for 72 h, and the size distributions were detected using DLS.

***In vitro* drug release**

The in vitro drug release from the polymeric nanoparticles was evaluated via a dialysis method. NC-NP or SA-NP were loaded into dialysis bags (MWCO 3500) and immersed in 50 mL PBS (10 mM) at pH 7.4 or 5.0 in a 37°C shaker (200 rpm). At timed intervals, 100 μL dialysate was withdrawn and replaced with an equal volume

of fresh medium. The platinum content was measured by ICP-MS, and the NCTD content was measured by HPLC.

Cellular uptake

The cellular uptake rate was quantified by measuring the intracellular Pt contents. 4T1 cells were seeded in 96-well plates at a density of 5 × 10^3 cells/well, incubated for 24 h, and then treated with free OXA, NC-NP, or SA-NP for 1 h, 2 h, or 6 h at a Pt-eq. dose of 3 μg/ml. The cells were washed three times with cold PBS and isolated to detect the Pt content by ICP-MS.

4T1 cells were seeded in glass-bottomed Petri dishes at a density of 1 × 10^5 cells per dish and incubated overnight. The medium was replaced with 1 mL of fresh medium containing $^{Cy5.5}$NC-NP or $^{Cy5.5}$SA-NP at a Cy5.5 dose of 0.5 μg/mL. After timed incubation (1h, 6h, or 12h), the cells were further incubated with LysoTracker Green (200 nM) for 30 min and Hoechst33342 (two drops) for 15 min. The medium was removed, and the cells were washed with cold PBS and then observed using CLSM. LysoTracker Green: ex, 488 nm; em, 500-550 nm. Hoechst 33342: ex, 405 nm; em, 425-475 nm. Cy5.5: ex, 640 nm; em, 680 nm.

We further determine the cell internalization pathway of NC-NP. 4T1 cells were seeded in 12-well plates at a density of 2×10^5 cells/well overnight. Then the cells were incubated under 4 °C or treated with endocytosis inhibitors chlorpromazine (50 μM), wortmannin(5 μM), cytochalasin D (5 μM), and filipin (7.5 μM) at 37 °C for 2 h and $^{Cy5.5}$NC-NP was added into wells. After 4 h incubation, the cells were washed with PBS three times, and the cellular uptake rate was examined by flow cytometry (BD FACSCalibur™, San Jose, CA)

In vitro cytotoxicity

In vitro cytotoxicity of polymeric nanoparticles was assessed using the 3-(4,5-Dimethylthiazolyl-2)-2,5-diphenyltetrazolium bromide (MTT) method. The cells were cultured in 96-well plates with a density of 5,000 cells per well for 24 h, followed by the addition of each formulation at a series of concentrations. After 48 h incubation, 20 μL of MTT solution (5 mg/mL) was added to incubate with cells for 4

h. The medium in each well was removed, and 100 μL of DMSO was added to dissolve the purple formazan crystals. The absorbance of the sample at 562 nm was detected using a microplate spectrophotometer (SpectraMax M2E, Molecular Device, USA). The cell survival rate is calculated by dividing the absorbance value of the experimental group by that of the control group. Each sample concentration was performed in triplicate, and three independent experiments were performed.

Live/dead cell analysis

4T1 cells were cultured in a 12-well plate at a density of 1 x 10^5 cells per well for 24 h. Then cells were treated with PBS, NC-NP, SA-NP, NCTD, OXA, and NCTD + OXA (NC-NP and SA-NP were pretreated at pH 7.4 or 5.0 for 24 h) for 48 h at a Pt-eq. dose of 3 μg/mL and NCTD-eq. dose of 5.2 μg/mL. Afterward, the culture medium was removed, and the cells were washed with PBS three times and stained with Live and Dead Cell Double Staining Kit (Calcein-AM and Propidium Iodide) at 37 °C for 15 min. Finally, the live and dead cells were observed under confocal fluorescence microscopy with 490 nm excitation.

PP2A phosphatase activity assay

4T1 cells were seeded in 6-well plates at a density of 2 × 10^5 cells per well and cultured overnight. After 6 h-incubation with PBS, NC-NP, SA-NP, NCTD, OXA, or NCTD +OXA at an NCTD-eq. dose of 5.2 μg/mL and OXA eq. dose of 3 μg/mL, the cells were washed twice with cold PBS and lysed in RIPA buffer containing protease inhibitor for 20 min (ice). Then the cell lysates were ultrasonic for 10 s and centrifuged for 15 min (1.5 × 10^4 g). The supernatants were collected and analyzed using the PP2A Immunoprecipitation Phosphatase Assay Kit.

Flow cytometric analysis of cell cycle

4T1 cells were seeded in 6-well plates at a density of 1.5 x 10^5 cells per well and cultured overnight, followed by treatment with different formulations at a Pt-eq. dose of 3 μg/mL and NCTD-eq. dose of 5.2 μg/mL. After 48 h, the cells were harvested and fixed with 70% ethanol overnight at 4°C. The fixed cells were incubated with RNase A (100 μg/mL) in PBS for 30 min at 37 °C and stained with PI (50 μg/mL) for

30 min in the dark before flow cytometry analysis. Each sample concentration was performed in triplicate, and three independent experiments were performed.

CRT analysis

4T1 cells were cultured in the 12-well plates (1×10^5 cells/well) overnight and then incubated with each formulation at a Pt-eq. dose of 3 μg/mL and NCTD-eq. dose of 5.2 μg/mL for 4 h. Then the cells were washed with PBS and fixed in 4% paraformaldehyde for 15 min. Afterward, the cells were washed with PBS and incubated with the anti-CRT antibody (ab196158, 1: 500) for 30 min. The CRT-positive cells were analyzed using flow cytometry.

ATP detection

Extracellular secretion of ATP was detected using the ATP detection kit. Briefly, 4T1 cells were cultured in 12-well plates (1×10^5 cells/well) for 12 h and then incubated with each formulation at a Pt-eq. dose of 3 μg/mL and NCTD-eq. dose of 5.2 μg/mL for 4 h. Afterward, the cell culture supernatant was collected, and the ATP contents were measured by the ATP detection kit according to the manufacturer's protocol.

HMGB1 analysis

4T1 cells were cultured overnight in the glass-bottomed Petri dishes at a density of 1×10^5 cells per dish and then incubated with each formulation at a Pt-eq. dose of 3 μg/mL and NCTD-eq. dose of 5.2 μg/mL for 24 h. The cells were washed with PBS three times and fixed in 4% paraformaldehyde for 15 min. The cells were permeabilized with 0.3% Triton X-100 for 15 min, followed by PBS washing and incubation with 5% BSA for 30 min. The cells were incubated with anti-HMGB1 for another 30 min and stained with DAPI for 15 min before CLSM observation.

DC maturation

Bone marrow-derived dendritic cells (BMDCs) were collected from the bone marrow of BALB/c mice and cultured in 1.5 mL 1640 medium containing GM-CSF (20 ng mL^{-1}) and IL-4 (10 ng mL^{-1}). 4T1 cells ($1 \text{ x } 10^5$ cells/well) and BMDCs (5×10^5 cells/well) were seeded in the transwells and 12-well plates, respectively, and

cultured overnight. 4T1 cells were cultured with different treatments for 24 h and then co-incubated with BMDCs for 24 h. Afterward, the BMDCs were harvested for antibody staining, and the matured DCs ($CD11c^+CD80^+CD86^+$) were analyzed using flow cytometry.

Flow cytometric analysis of PD-L1

4T1 cells were cultured in the 12-well plates at a density of 1×10^5 cells per well overnight and then incubated with each formulation at a Pt-eq. dose of 3 μg/mL and NCTD-eq. dose of 5.2 μg/mL for 24 h. Then the cells were washed with PBS and incubated with the anti-PD-L1 antibody (1: 500) for 30 min. The PD-L1-positive cells were analyzed using flow cytometry.

Western blotting analysis

For western blotting analysis, the cells with different treatments were lysed with RIPA cell lysis buffer. The protein concentration was determined by the BCA protein detection kit, and samples containing 40 μg protein were loaded onto an SDS-PAGE gel (8% separating gel). The proteins in the cell lysates were separated and electrotransferred to a PVDF membrane. The membrane was then blocked with 5% non-fat powdered milk in TBST buffer for 1 h, followed by incubation with primary antibodies: β-Catenin (1: 1000), PD-L1 (1: 1000), or GAPDH (1: 1000) at 4 °C overnight. The membrane was washed with TBST three times and incubated with horseradish peroxidase-labeled goat anti-rabbit or goat anti-mouse secondary antibody (1: 1000) at room temperature for 1 h. Afterward, the membrane was washed with TBST three times and visualized with a chemiluminescence imaging system (CLiNX Science instruments, China). The grey value ratios of the bands to the internal reference were analyzed by ImageJ software to obtain the relative protein expressions.

Blood Clearance

Female ICR mice (6-8 weeks) were intravenously (*i.v.*) injected with free OXA, NC-NP, or SA-NP at a Pt-eq. dose of 1 mg/kg (n=3). At timed intervals, 50 μL blood samples were collected from the orbital venous plexus of the mice, heparinized, and centrifuged (5000 rpm, 10 min) to obtain the plasma. The samples were decomposed

in aqua regia, and the Pt contents were detected by ICP-MS.

Biodistribution

Female BALB/c mice (6-8 weeks) were subcutaneously injected with 5 × 10^5 4T1 cells into the mammary fat pad. When the tumor volume reached ~ 80 mm^3, the mice were randomly divided into 3 groups (n=3) and *i.v.* injected with free OXA, NC-NP, or SA-NP at a Pt-eq. dose of 1 mg/kg. After 24 h postinjection, the mice were sacrificed, and tumors and main organs, including the heart, liver, spleen, lung, and kidneys, were collected. The Pt contents were measured by ICP-MS.

***In vivo* antitumor activity**

Female BALB/c mice (6-8 weeks) bearing orthotopic 4T1 tumors of ~ 100 mm^3 were randomly divided into six groups (n=7), and i.v. injected with PBS, OXA, NCTD, OXA+NCTD, SA-NP, or NC-NP at a Pt-eq. dose of 1 mg/kg and NCTD-eq. dose of 1.7 mg/kg every two days three times. The body weight and tumor volume were recorded individually. On day 22 post-treatment, the mice were sacrificed according to animal ethical requirements, and tumors and major organs were collected. Tumor volume was calculated according to the formula: $V = 0.5 \times LW^2$ (L: tumor length, W: tumor width). The tumor inhibition rate (TIR) was calculated following the formula: TIR = 100% × (mean tumor weight of control group - mean tumor weight of experimental group)/ mean tumor weight of control group.

***In vivo* antitumor immune response**

Female BALB/c mice (6-8 weeks) bearing orthotopic 4T1 tumors of ~ 100 mm^3 were randomly divided into six groups (n=3), and i.v. injected with PBS, OXA, NCTD, NCTD + OXA, SA-NP, or NC-NP at a Pt-eq. dose of 1 mg/kg and NCTD-eq. dose of 1.7 mg/kg every two days for 3 times. The mice were sacrificed 7 days after the last administration, and blood, tumors, and tumor-draining lymph nodes (TDLNs) were collected. Blood samples were used to analyze the systemic cytokines, including interferon-γ (IFN-γ), tumor necrosis factor-α (TNF-α), interleukin-2 (IL-2), interleukin 10 (IL-10), and interleukin 12 (IL-12) using the corresponding ELISA kit according to the manufacturer's protocol.

The collected TDLNs were ground and centrifuged to obtain cell suspensions. DCs were treated with APC anti-CD11c, PE anti-CD80, and FITC anti-CD86 antibodies and analyzed using flow cytometry.

The tumors were divided into two parts. One portion of the tumors was sliced into approximately 8-μm-thick sections and fixed with 4% paraformaldehyde for 15 min, followed by PBS washing and incubation with blocking solution (1% BSA, 0.5% Triton X-100 in PBS) for 30 min. Then the sections were stained with CRT primary antibodies (1: 100) and PD-L1 primary antibodies (1: 100) overnight at 4°C, followed by incubation with Alexa Fluor 488 or Alexa Fluor 647-conjugated secondary antibodies (1: 200) for 1 h at room temperature. After staining the nuclei with DAPI, the sections were observed using CLSM.

Another portion of tumors was homogenized to obtain a cell suspension. The cell suspensions were stained with PerPC anti-CD3, FITC anti-CD4, and PE anti-CD8a antibodies to analyze effector T cells; Homogenized tumor cell suspensions were stained with PerPC anti-CD3, FITC anti-CD4, and PE anti-FOXP3 antibodies for Tregs analysis.

Histological analysis

H&E assay: The major organs (heart, liver, spleen, lungs, and kidneys) and tumors were fixed with 4% neutral-buffered paraformaldehyde for 48 h and then dehydrated with gradient alcohol. The tissues were embedded with paraffin and sliced into thickness 4-μm-thick sections. The slices were stained with hematoxylin and 1% eosin solution and then observed under an optics microscope.

TUNEL assay: The fresh tumor tissues harvested from the sacrificed mice were fixed with 4% neutral-buffered paraformaldehyde, embedded in paraffin, and sectioned into 4-μm-thick slices. The tumor sections were treated with a TUNEL apoptosis assay kit, according to the manufacturer's protocol. Apoptotic cells in each section were imaged by a confocal microscope.

Ki67 assay: The harvested tumor and kidney tissues were fixed with 4% neutral-buffered paraformaldehyde, embedded in paraffin, and sectioned into

4-μm-thick slices. Then the slices were treated with Ki-67 Detection KIT according to the manufacturer's protocol and observed using fluorescent microscopy.

Statistical analyses

Statistical analysis was performed using GraphPad Prism and Excel. The two-tailed, unpaired Students' t-test was utilized to calculate the significance. Data are presented as mean ± SD. $P < 0.05$ was regarded as statistically significant.

Supplementary Figures

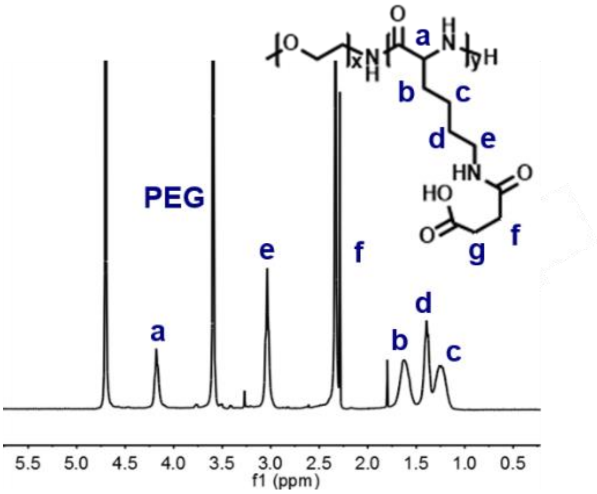

Figure S1. The ^{1}H NMR spectrum of PEG-PLL/SA in D_2O.

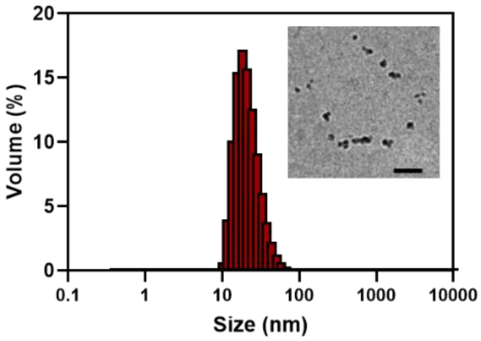

Figure S2. The size distribution of SA-NP analyzed by DLS and the morphologies of SA-NP observed by TEM, scale bar = 200 nm.

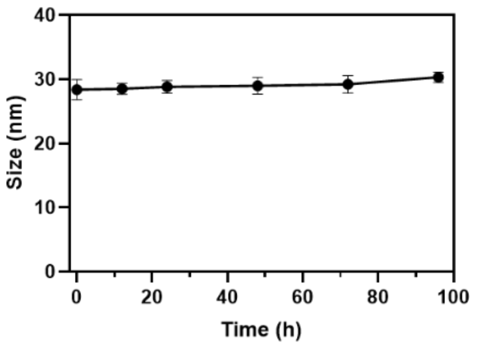

Figure S3. The size variation of NC-NP during storage at 37 °C.

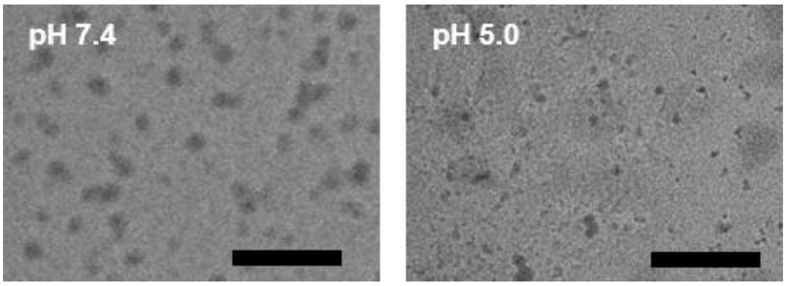

Figure S4. The TEM images of NC-NP after incubation at pH 7.4 or 5.0 for 72 h. scale bar = 200 nm.

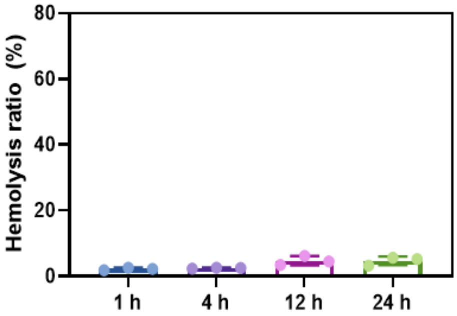

Figure S5. The hemolysis result of NC-NP at pH 7.4.

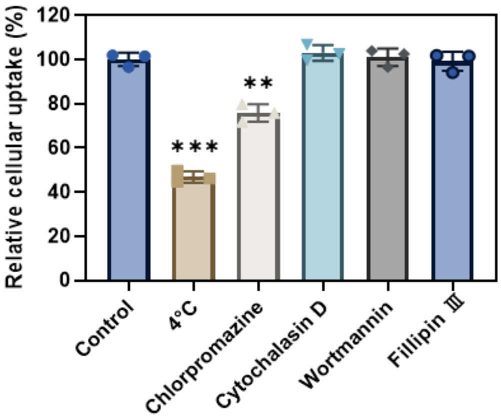

Figure S6. The relative cellular uptake of NC-NP in 4T1 cells in the presence of endocytic inhibitors.

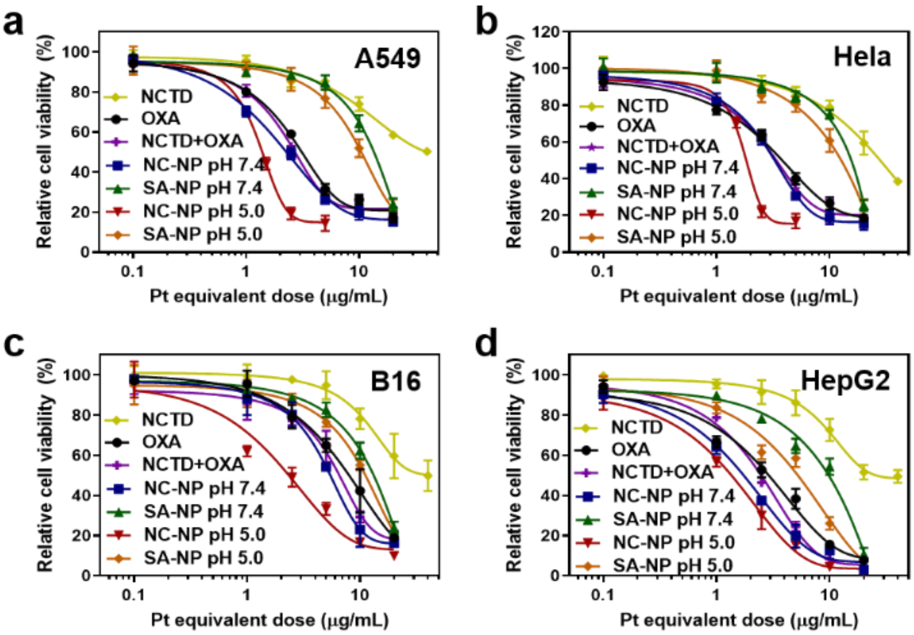

Figure S7. The in vitro cytotoxicity of NC-NP against A549, Hela, B16, or HepG2 cells at pH 7.4 or 5.0 determined by MTT assay.

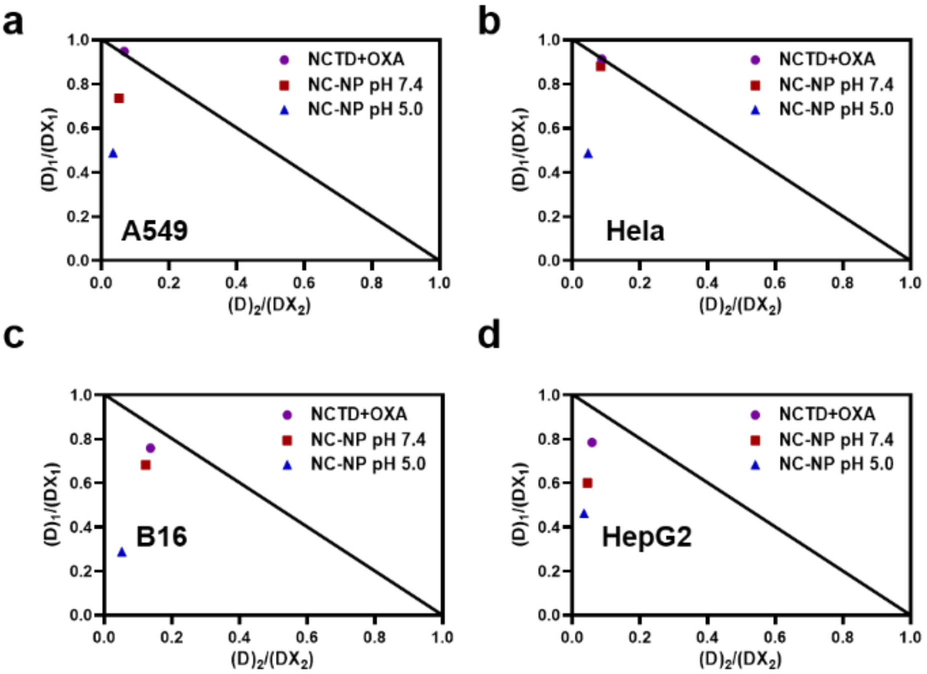

Figure S8. The combination index of NCTD and OXA in NC-NP against A549, Hela, B16, or HepG2 cells.

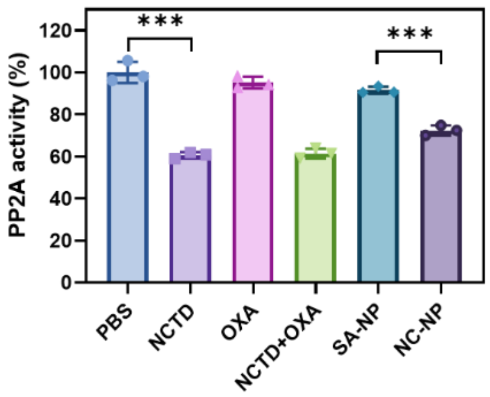

Figure S10. PP2A activity of B16 cells after 6 h incubation with each formulation at an OXA eq. dose of 3 μg/mL and NCTD-eq. dose of 5.2 μg/mL.

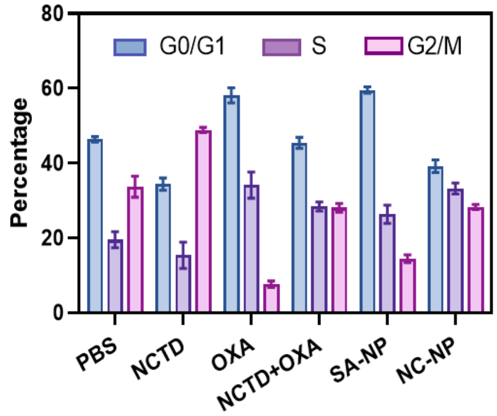

Figure S11. Quantitative analysis of cell cycle distribution of 4T1 cells after each treatment in Figure 2h (n=3).

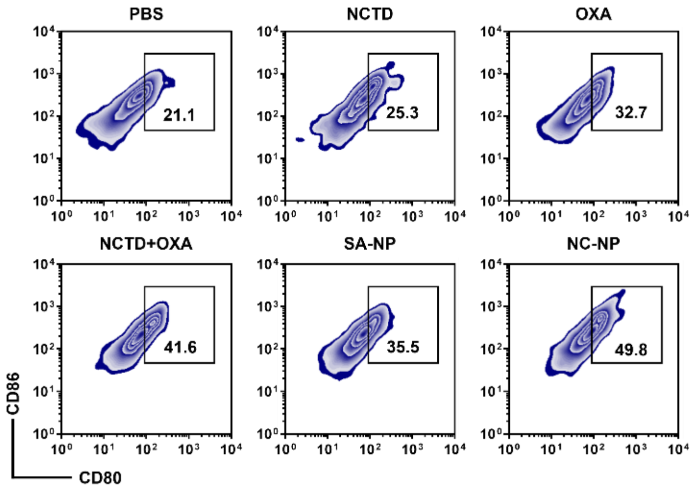

Figure S12. Typical flow cytometry analysis profiles of matured DCs (gated on $CD80^{+}CD86^{+}$) induced by each formulation.

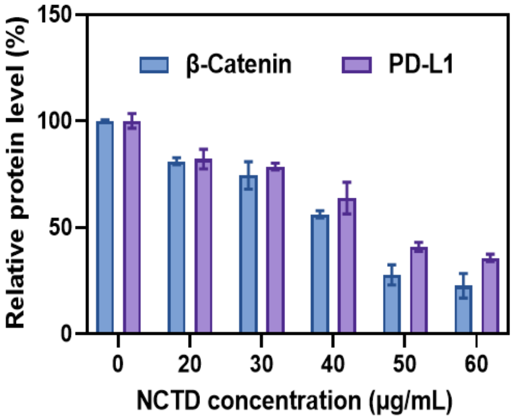

Figure S13. Quantification of the western blotting results in Figure 3i.

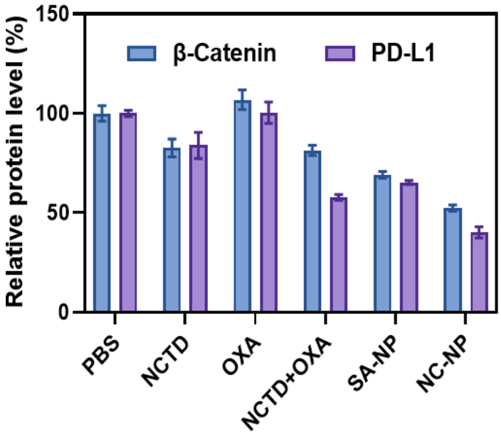

Figure S14. Quantification of the western blotting results in Figure 3j,l.

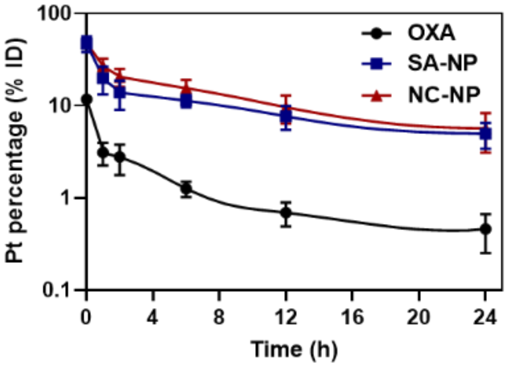

Figure S15. Blood clearance kinetics of OXA, SA-NP, and NC-NP (OXA-eq. dose, 1

mg/kg; n = 3)

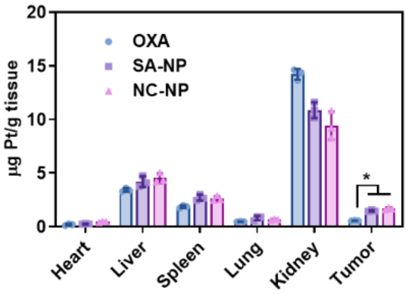

Figure S16. Biodistributions of OXA, SA-NP, and NC-NP at 24 h postinjection, OXA-eq. dose, 1 mg/kg; n = 3.

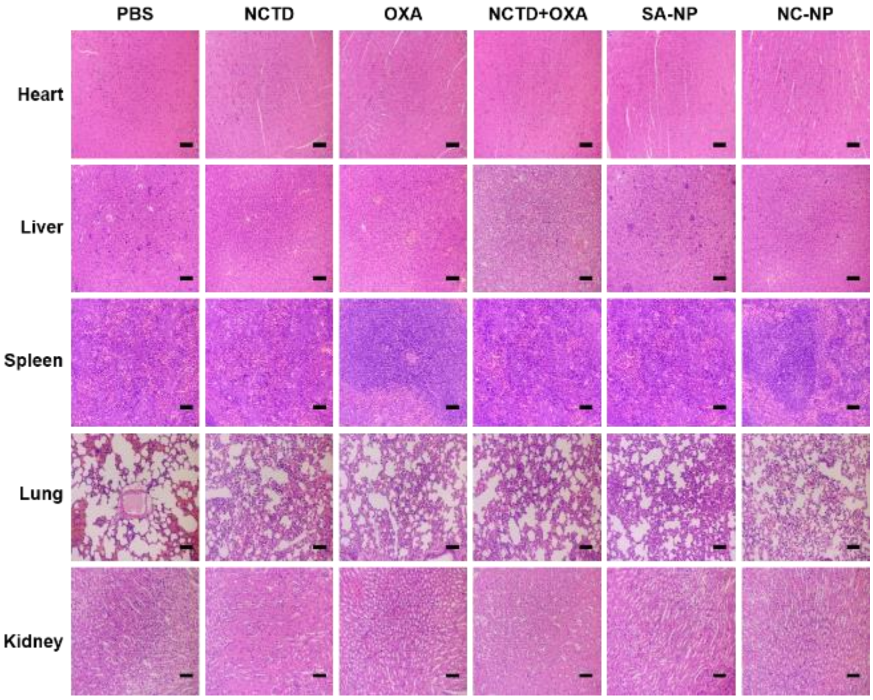

Figure S17. Representative histological features of organs (liver, lung, spleen, kidney, and heart) from the mice bearing 4T1 tumors treated with PBS, NCTD, OXA, NCTD + OXA, SA-NP, or NC-NP. The 4-µm-thick tissue paraffin sections were stained with hematoxylin-eosin and observed by light microscopy. Scale bars are 250 µm.

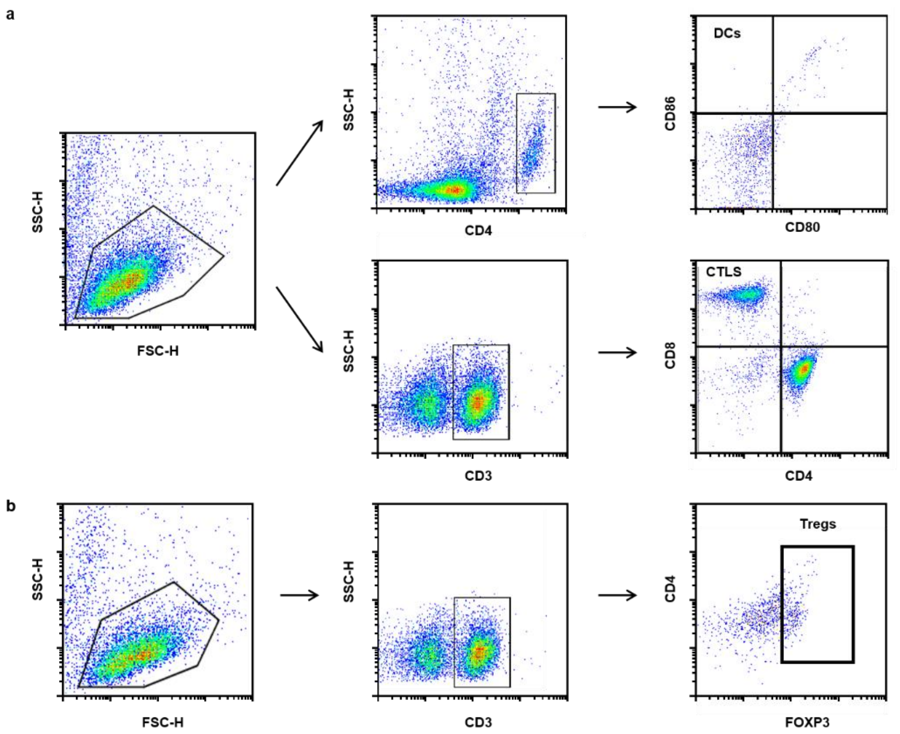

Figure S18. Gating strategies for flow cytometry. (a) Gating strategy to identify mature DCs and cytotoxic T lymphocytes presented in Figures 5a, c, k, and m; (b) Gating strategy to identify regulatory T cells presented in Figure 5c and o.

Supplementary tables

Table S1. IC_{50} values of each formulation against various cell lines, 48 h treatments (Units: µg/mL).

Cell lines	NCTD	NCTD+OXA	OXA	NC-NP pH 7.4	SA-NP pH 7.4	NC-NP pH 5.0	SA-NP pH 5.0
4T1	39.91	2.48	3.03	3.22	13.52	1.97	8.95
B16	39.62	6.30	8.29	5.66	13.19	2.39	11.53
HepG2	34.67	2.34	2.98	1.79	10.98	1.38	6.311
A549	40.21	3.14	3.31	2.44	13.53	1.62	6.46
Hela	35.56	3.60	3.94	3.47	15.04	1.92	12.89

Table S2. Combination index (IC) of NCTD and OXA in each formulation against various cell lines.

Cell lines	NCTD+OXA	NC-NP pH7.4	NC-NP pH 5.0
4T1	0.87	1.13	0.69
B16	0.90	0.80	0.34
HepG2	0.84	0.64	0.50
A549	1.01	0.79	0.52
Hela	1.00	0.96	0.53

Table S3. Pharmacokinetic parameters of OXA. SA-NP, and NC-NP at a OXA eq. dose of 1 mg/kg.

Parameter	OXA	SA-NP	NC-NP
$T_{1/2}\,\alpha$ (h)	0.049	0.40	0.49
$T_{1/2}\,\beta$ (h)	4.7	15.2	11.7
AUC (ID%·h)	31.0	232.7	299.2
